# Antiseptic Treatment of Scarlet Fever

**Published:** 1891-10-10

**Authors:** J. Brendon Curgenven

**Affiliations:** Teddington Hall


### Editor's Letter-Box.

[Our correspondents are reminded that prolixity is a great bar to publication, and that brevity of style and conciseness of statement greatly facilitate early insertion].

### ANTISEPTIC TREATMENT OF SCARLET FEVER.

Sir,—On March 29th in last year you favourably noticed the paper which I read before the Epidemiological Society on the 12th of that month on the treatment and disinfection of scarlet fever by eucalyptus. That paper has since been published by Mr. Lewis, of Gower-street. It may be remembered that I proved, by the relation of several cases treated by inunction of the Eucalyptus disinfectant, that not only did the fever rapidly subside, the temperature falling two or three degrees after the first inunction, but also that the power of infection was completely arrested, the patients required no isolation, and could join the rest of their family when convalescent, or in about ten days. Further experience, and the experience of other medical men, confirms all I then stated, and I have given the results in a paper which I read before the International Congress of Hygiene, and which will shortly be published. This system of treatment and disinfection has been adopted in several public schools and other institutions, as it speedily stops any outbreak of infectious disease. In one public school of 200 resident boys the severity of the fever in the boys attacked was considerably modified by the inunction, and the outbreak stopped. Dr. Peake, of Shepherd's Bush, first treated his own child by this method, and placed his two other children in the same room and kept them there for eight days; at the end of that time the child was convalescent, and the other two did not take the disease. I will not take up more of your space by further quotations, as I have gone fully into the subject in my papers, but I think the eucalyptus treatment will bear a favourable comparison with that by biniodide of mercury, which does not arrest infection, and through the use of which evil results to the kidneys might ensue.—Yours faithfully, J. Brendon Curgenven.

Teddington Hall, October 3rd, 1891.

